# Selected abstracts of “Bioinformatics: from Algorithms to Applications 2020” conference

**DOI:** 10.1186/s12859-020-03838-2

**Published:** 2020-12-17

**Authors:** 

## I1

### Fourth International Conference “Bioinformatics: From Algorithms to Applications” (BiATA 2020)

#### Alla Lapidus^1,*^, Anton Korobeynikov^1^

##### ^1^Center for Algorithmic Biotechnologies, Saint Petersburg State University, Saint Petersburg, Russia, 199034

**Correspondence**: Alla Lapidus - a.lapidus@spbu.ru

*BMC Bioinformatics* 2020, **21(Suppl 20)**: I1

International Conference “Bioinformatics: from Algorithms to Applications” (BiATA) is one of the few international conferences that bring together both the programmers and algorithm developers creating tools for a wide spectrum of modern bioinformatics studies and the researchers conducting those experiments interested in finding reliable and easy to use tools for data analysis.

COVID-19 this year forced conferences online. Virtual conferences offer new experience that on a positive side allowed more participants to attend meetings that would be too hard or too expensive to attend in person. That's exactly what happened to BiATA-2020 that we had had to run remotely this year: we had a pleasure to welcome 475 scientists from all over the world versus 100 participants in previous years!

Follow our traditions, BiATA2020 promotes active application of bioinformatics in numerous fields of research, identifies new trends in the fields of bioinformatics, computational genomics and transcriptomics, as well as in discovery of biologically active molecules.

Topics covered within the framework of the conference include but are not limited to:Sequencing technologiesMolecular sequence analysisComputational genomicsGenome assemblyTranscriptomicsMetagenomicsAgrigenomicsViromicsNatural Products Discovery
The Fourth international conference “Bioinformatics: from Algorithms to Applications” was held on July 27–28, 2020 and was accompanied by the 2-day online workshop that included

metagenomic data analysis and annotation using MGnify [1].

**Reference**Mitchell AL, et al., MGnify: the microbiome analysis resource in 2020. Nucleic Acids Res. 2020 Jan 8;48(D1):D570–D578. 10.1093/nar/gkz1035

## O1

### VarQuest+: modification-tolerant database search of secondary metabolites mass spectra

#### Azat M. Tagirdzhanov^1,2^, Egor Shcherbin^3^, Hosein Mohimani^4^, Alexey Gurevich^1*^

##### ^1^Center for Algorithmic Biotechnology, St. Petersburg State University, St. Petersburg, Russia; ^2^Department of Higher Mathematics, St. Petersburg Electrotechnical University “LETI”, St. Petersburg, Russia; ^3^National Research University Higher School of Economics, Moscow, Russia; ^4^Computational Biology Department, School of Computer Sciences, Carnegie Mellon University, Pittsburgh, PA, USA

**Correspondence**: Alexey Gurevich - aleksey.gurevich@spbu.ru

*BMC Bioinformatics* 2020, **21(Suppl 20)**: O1

Secondary metabolites (SMs) are at the center of attention for a wide range of researchers from biologists and ecologists to pharmacologists and biomedical scientists [1]. Modern mass spectrometry instruments allow rapid and low-cost scanning of thousands of metabolites which result in huge amounts of high-resolution data. Although this data represents a gold mine for future discoveries, its interpretation remains a bottleneck and requires appropriate computational methods [2]. The current software is either limited to specific classes of SMs, for example, peptidic natural products (VarQuest [3]), or can perform only standard database search which allows identification of known SMs but fails to discover their novel variants (Dereplicator + [4]).

Here we present VarQuest+, a database search tool capable of identifying novel variants of a wide range of known SMs including polyketides, alkaloids, flavonoids, saponins, and many others. Algorithmic and software innovations in VarQuest + make it much more efficient in the running time and memory consumption in comparison to existing analogs. This efficiency allowed the implementation of modification-tolerant search mode in VarQuest+, which is more challenging than a regular database search.

We benchmarked VarQuest + on a Korean medical plants dataset (2.5 millions of mass spectra collected on 337 samples). The standard search of the KNApSAcK database (51,179 plant SMs [5]) resulted in the identification of 349 compounds. VarQuest + modification-tolerant search identified 4253 SMs, an order of magnitude more than Dereplicator + . Using the same search parameters, VarQuest + is twenty times more efficient than Dereplicator + in runtime, and four times more memory efficient.

The reported study was funded by RFBR, project number 20-04-01096.

## References


Cragg GM, Newman DJ. Natural products: a continuing source of novel drug leads. Biochim Biophys Acta. 2013; 1830(6):3670–3695.Wang M, Carver JJ, Phelan VV, et al. Sharing and community curation of mass spectrometry data with Global Natural Products Social Molecular Networking. Nat Biotechnol. 2016; 34(8):828–837.Gurevich A, Mikheenko A, Shlemov A, Korobeynikov A, Mohimani H, Pevzner PA. Increased diversity of peptidic natural products revealed by modification-tolerant database search of mass spectra. Nat Microbiol. 2018; 3(3):319–327.Mohimani H, Gurevich A, Shlemov A, et al. Dereplication of microbial metabolites through database search of mass spectra. Nat Commun. 2018; 9(1):4035.Afendi FM, Okada T, Yamazaki M, et al. KNApSAcK family databases: integrated metabolite-plant species databases for multifaceted plant research. Plant Cell Physiol. 2012; 53(2):e1.

## O2

### Genome-wide inference of bacterial transcription factor binding sites: new method and its applications

#### Yevgeny Nikolaichik^1*^, Pavel Vychik^1^

##### ^1^Department of Molecular Biology, Belarusian State University, Minsk, Belarus

**Correspondence**: Yevgeny Nikolaichik - nikolaichik@bsu.by

*BMC Bioinformatics* 2020, **21(Suppl 20)**: O2

**Background** Transcription factor binding sites (TFBS or operators) are the most abundant regulatory elements in genomes. Locating them is critical for understanding genome expression, but the methods for their automated genome-wide inference are lacking.

**Materials and methods** Our method of bacterial TFBS inference extends an idea [1] of extracting relevant information from 3D structures of transcription factor (TF)-operator complexes. We use TF residues contacting DNA bases as a tag (CR-tag) to link TFs with their operators. Calibrated CR-tagged TFBS profiles are used for automated genome-wide operator inference.

GUI application implementing this method was developed in Xojo with some routines written in python. RegPrecise was used as the major source of TFBS data [2].

**Results** TFBSs are inferred genome-wide via either (1) fast automated CR-tag based genome scan with a library of CR-tagged experimentally characterised TFBS motifs or (2) slow semi-automated de novo TFBS inference combining CR-tag information with genome structure analysis. The first approach allows to reliably transfer regulatory information between different species. The de novo protocol resembles phylogenetic footprinting approach, but replaces orthology assumptions by strict 3D-structure based criterium (CR-tag).

The method can be used for:Correcting poorly defined motifs.For most TFs in a given species, just one or very few targets exist and proper TFBS models cannot be built. With the de novo TFBS inference protocol, sufficient number of orthologous operator sequences can be collected from other species that have TFs with identical CR-tag allowing proper definition of the operator motif and building its high-quality model. This approach can vastly improve the usability of the data from single-organism TFBS databases.Resolving regulation details for paralogous TFs.Using our CR-tag based approach and experimental evidence, we investigate a case of completely different operators reported for paralogous TFs.Correcting automated genome annotation.Finding an operator for a well-characterised TF can suggest functions for the downstream genes [3].Full-scale genome-wide TFBS inference.With a current collection of TFBS profiles, genome-wide scan finds operators for the majority of operons in a typical enterobacterial genome. This helps to reveal unexpected regulators for many genes and allows deciphering regulatory cascades.**Conclusions** The CR-tag based TFBS inference method is applicable to any bacterium and can find the majority of TFBSs in uncharacterised bacterial genomes. The application implementing the method and its source code are freely available at github.com/nikolaichik/SigmoID.

**References**Sahota G, Stormo GD. Novel sequence-based method for identifying transcription factor binding sites in prokaryotic genomes. Bioinformatics. 2010;26:2672–7.Novichkov PS, Kazakov AE, Ravcheev DA, Leyn SA, Kovaleva GY, Sutormin RA, et al. RegPrecise 3.0—A resource for genome-scale exploration of transcriptional regulation in bacteria. BMC Genomics. 2013;14:745.Nikolaichik Y, Damienikan AU. SigmoID: a user-friendly tool for improving bacterial genome annotation through analysis of transcription control signals. PeerJ. 2016;4:e2056.

## O3

### A platform for genomic characterization of *Enterococcus* spp.

#### Ícaro M. S. Castro^1*^, Janira Prichula^2^, Rafaella S. Bueno^2,3^, Robson D. Ruiz^2,3^, Adriana Seixas^3^

##### ^1^Institute of Mathematics and Statistics (IME), University of São Paulo (USP), São Paulo, São Paulo, Brazil; ^2^g-positive Cocci Laboratory, Federal University of Health Sciences of Porto Alegre (UFCSPA), Porto Alegre, Rio Grande do Sul, Brazil; Department of Pharmacosciences, UFCSPA, Porto Alegre, Rio Grande do Sul, Brazil

**Correspondence**: Ícaro M. S. Castro - icaromscastro@gmail.com

*BMC Bioinformatics* 2020, **21(Suppl 20)**: O3

**Background** Enterococci have emerged as one of the main bacterial genera of clinical relevance. Genomic studies have greatly contributed to the understanding of biology, virulence, and evolution of *Enterococcus* spp. The growing demand for genomic data analysis made essential the development of scalable and robust bioinformatic workflows. Until the moment, there are few genomic analysis workflows designed for a specific bacterial genus. Here, we propose JAMIRA—a reproducible and scalable workflow for prokaryote genomic data analysis designed for the genera *Enterococcus* spp.

**Materials and methods** Benchmark bioinformatic tools were compared in order to define the most appropriate tools for the genus *Enterococcus* spp. To ensure data analysis reproducibility, JAMIRA workflow was designed using the Snakemake framework. Bioinformatic tools were installed in isolated environments with Anaconda package manager to encapsulate all software dependencies. JAMIRA includes the following bioinformatic tools: Abricate for virulence gene prediction, RGI for antimicrobial resistance gene prediction, PlasmidFinder for plasmid prediction, IslandPath-DIMOB for genomic islands prediction, PhiSpy for prophage prediction and Prokka for genome annotation. A web application of JAMIRA is being implemented using the PHP Laravel framework for the elaboration of the program's internal structure and JavaScript, HTML, and CSS for graphical user interface.

**Results** JAMIRA automates several bioinformatic analyses commonly performed in comparative genomic studies in order to elucidate the biological mechanisms which associate enterococci strains with public health outcomes. The application has a graphical interface that allows the submission of genomic data in FASTA file format, dispensing manual software installation, previous genome annotation, and the use of a command-line interface. Besides, JAMIRA is an interesting platform for the scientific community, since it reduces the researcher’s computational efforts and also ensures data analysis reproducibility.

**Conclusions** JAMIRA is an automated and easy-to-use workflow that can allow scientists with no background in bioinformatics to perform reproducible genomic data analyses. The proposed workflow might contribute as a state art methodology to design bioinformatics workflows for specific genus as well as contributing to the understanding of the role implied by commensal and clinical enterococci in different environments and to promote elucidation of biological mechanisms which made this bacterial genus associated with public health risks.

## O4

### Do multiple long-distance transfers shape TBEV spread pattern?

#### Andrei A. Deviatkin^1,2*^, Yulia A. Vakulenko^3,4^, Ivan S. Kholodilov^5^, Galina G. Karganova^5,6^, Alexander N. Lukashev^1,3^

##### ^1^Laboratory of Molecular Biology and Biochemistry, Institute of Molecular Medicine, Sechenov First Moscow State Medical University, 119048 Moscow, Russia; ^2^Laboratory of Postgenomic Technologies, Izmerov Research Institute of Occupational Health, 105275 Moscow, Russia; ^3^Martsinovsky Institute of Medical Parasitology, Tropical and Vector Borne Diseases, Sechenov First Moscow State Medical University, 119435 Moscow, Russia; ^4^Department of Virology, Faculty of Biology, Lomonosov Moscow State University, 119234 Moscow, Russia; ^5^Laboratory of Biology of Arboviruses, Chumakov Institute of Poliomyelitis and Viral Encephalitides (FSBSI “Chumakov FSC R&D IBP RAS), 108819 Moscow, Russia; ^6^Department of Organization and Technology of Immunobiological Preparations, Institute for Translational Medicine and Biotechnology, Sechenov First Moscow State Medical University, 119991 Moscow, Russia

Correspondence: Andrei A. Deviatkin - andreideviatkin@gmail.com

BMC Bioinformatics 2020, **21(Suppl 20)**: O4

Tick-borne encephalitis (TBE) is viral zoonosis transmitted by the bite of infected ticks. In 1999, phylogenetic analysis demonstrated clear separation of TBE viruses into three subtypes, that were called based on its distribution: European, Siberian, and Far-Eastern. It is now becoming apparent that the actual spread of these viruses may differ from the nominal. Herein, 848 TBEV sequences (1028 nt E-gene fragments) were analyzed to indicate all long-distance virus transfers, that can be revealed from the sequence data. Threshold of 500 km was used for the selection of long-distance virus transfers. Noteworthy, ticks are not able to spread the infection on their own over such a distance. In other words, these long-distance virus transmissions were caused by vector-assisted tick transmission. In all subtypes and most of the smaller groups in these subtypes, there were a lot of recent long-distance virus transfers, that was revealed by Bayesian evolutionary analysis. Moreover, this is suggested to be a systematic pattern, rather than anecdotal events. For example, 19 out of 125 known sequences the Far-Eastern subtype were obtained in Japan. Genetic diversity of viruses found within this country was comparable with the diversity of the whole subtype. At the same time, this subtype is distributed throughout Japan, China, South Korea, Russia, Estonia and Latvia. The above arguments allow us to state that long transfers may be considered as a normal and abundant pattern in TBEV spreading.

**Acknowledgements**

This research was funded by the Russian Science Foundation (Grant # 19-75-00013).

## O5

### A rigorous approach to pairwise distance analysis of a protein family via multi-dimensional scaling and its application to the genealogy of squalene synthase paralogues of green algae

#### Robert B. Moore*^1^, Michael Barnathan^2†^, Brian Fristensky^3†^, Yan Li^4, 5†^, Gregory Knowles^4†^, Paul Gardner-Stephen^4^, Angelo Bueti^4^, Peter Anderson^4^, Jianguang Qin^4^, and Andrew S Ball^1^

##### ^1^School of Science, RMIT University, Bundoora, Victoria 3083, Australia; ^2^Temple University, Philadelphia, Pennsylvania 19122, USA; ^3^Department of Plant Science, University of Manitoba, Winnipeg, Manitoba R3T 2N2, Canada; ^4^School of Biological Sciences, Flinders University, Bedford Park, South Australia 5042, Australia; ^5^Norwegian Institute for Water Research, Oslo NO-0349, Norway (current address)

Correspondence: Robert B. Moore - science@academicmail.org

*BMC Bioinformatics* 2020, **21(Suppl 20)**: O5

^†^Michael Barnathan, Brian Fristensky, Yan Li and Gregory Knowles authors contributed equally to this work.

**Background** Gene duplication resulting in paralogues is an imperfectly understood process in terms of the order of duplications facilitating the evolution of new functions. An example is the Squalene Synthase-Like (SSL) gene family of the oil-bearing green alga *Botryococcus braunii* race B. Squalene synthases combine two half reactions of catalysis, being the formation of presqualene disphosphate (PSPP) and then the subsequent synthesis of a triterpenoid, in this case squalene. It was previously established that the SSL paralogues have separated these two half reactions.

**Materials and methods** The squalene synthase (SS) and SSL genes of the organism were sequenced using Illumina reads and SOAP and Velvet assembly, in such a way that the rest of the genome was essentially ignored but this gene family was retrieved in complete detail. By this means the full set of paralogues were determined. Secondly, the genetic “distances” between four genes (as in-silico proteins) so recovered were compared pairwise with each other, as well as with the same set of genes published by a previous group, and with other green algal SS genes. Finally, the pairwise distance comparisons were input into a novel algorithm for Multi-Dimensional Scaling (MDS), which in combination with standard substitution matrices and a simple averaging model for evolutionary rate of the genes, enabled a tree to be derived. Specifically, 5 dimensions were used in the MDS.

**Results** The order of evolution SS → SSL2 → SSL1 /SSL3 was determined, and inference of an evolutionary scenario was made. Further, an alignment process necessitated reannotation of key squalene synthases from green algal model organisms *Chlamydomonas* and *Volvox*, with support also obtained from the homologous proteins of non-model green algae *Micromonas* and *Ostreococcus*.

**Conclusions** Gene *B. braunii* SS, also known as BSS, has diverged further than a typical green algal SS, because selection on BSS was relaxed through duplication to create SSL2 which has all the functions of BSS except that PSPP synthesis is down-regulated without disappearing. C-terminal analysis indicated that green algal SSs may be membrane associated via two transmembrane alpha-helices plus an additional putative anchoring region. By this C-terminal indicator, BSS and SSL2 proteins may be in the same compartment/organelle as each other, explaining the somewhat relaxed selection on each. By contrast SSL1 and SSL3 which together generate a triterpenoid isomer named botryococcene, are lacking the C-terminal transmembrane alpha helices when analysed bioinformatically. This may indicate they have migrated to a different compartment or are in some way separated from the site of squalene synthesis, that has facilitated their separate evolution. SSL1 and SSL3 have stochastically recombined with each other which may have also facilitated the evolution of their new combined function—botryocccene synthesis, and are predicted to exist as a heterodimer.

## O6

### Black cat in a dark room: search for new viruses in metagenomes

#### Yulia Yakovleva^#1,2*^, Alexey Zabelkin^#3^, Maria Skazina^2,4^, Artyom Kaltovich^2^, Dmitry Antipov^5,6^, Mikhail Rayko^5,6^

##### ^1^Department of Cytology and Histology, Saint Petersburg State University, Saint Petersburg, Russia; ^2^Bioinformatics Institute, Saint Petersburg, Russia; ^3^ITMO University, Saint Petersburg, Russia; ^4^Department of Applied Ecology, Saint Petersburg State University, Saint Petersburg, Russia; ^5^Center for Algorithmic Biotechnology, Saint Petersburg State University, Saint Petersburg, Russia; ^6^Institute of Translational Biomedicine, Saint Petersburg State University, Saint Petersburg, Russia

Correspondence: Yulia Yakovleva - st041958@student.spbu.ru

*BMC Bioinformatics* 2020, **21(Suppl 20)**: O6

^#^Yulia Yakovleva and Alexey Zabelkin have contributed equally.

Detection of hidden viral diversity is a challenging task, which goes beyond the standard protocol of processing metagenomic data. Meanwhile, publicly available databases contain a large amount of metagenomic data—the promising source of novel viral genomes, which remains largely understudied. Here we present the new pipeline for detecting full-length viral genomes from assembled metagenomes.

Viral genomes represent cyclic or linear molecules with the ends containing repeated sequences. Both types could be recognized as cyclic sequences. We detect such contigs by searching repeats ranging from 50 to 200 bp using Knuth-Morris-Pratt algorithm. This algorithm takes linear time depending on the maximum length of the allowed repeat, which permits to process large amounts of data and reduce its dimensionality. We classify cyclic sequences as viral or non-viral based on predicted gene content using viralVerify tool. For each selected viral contig we identify the capsid and terminase genes based on HMM profiles. We aligned found protein sequences against the NCBI nr database with Diamond. The protein sequences, both queries and hits, belonging to each HMM profile were clustered with CD-HIT v4.8.1 (span 80%, identity 50%). The resulting centroid sequences were aligned using MAFFT v7.310 with default parameters, followed by phylogeny reconstruction using UPGMA and RAxML v8.2.11 separately. Clusters that do not contain any hits were classified as previously unknown. The completeness of viral contigs was inspected with viralComplete and CheckV. We tested our pipeline on assembled metagenomes from NCBI Assembly database. More than 170 Gb of data representing about 1300 metagenomes derived from seawater, soil and biofilms habitats were analyzed.

Our analysis revealed that the diversity of viruses is much greater than we know up to date. Hundreds of new viruses clusters were detected. For example, we identified 3 new representatives of the Siphoviridae and Podoviridae bacteriophage families from 10 biofilm-derived metagenomes. Our approach allows us to detect full-length viral genomes with a lower chance of false-positive results. In the future, the user of our pipeline can submit metagenome assemblies or raw reads to the input and receive annotated viral genomes from the data. Further analysis of metagenomes from other habitats is indispensable.

Project is available on GitHub: https://github.com/Yulia-Yakovleva/metavirome.

**Acknowledgments**

This work was supported by Saint Petersburg State University (project ID 51555639).

## O7

### The 3C criterion: Contiguity, Completeness and Correctness to assess de novo genome assemblies

#### Jose Arturo Molina-Mora^1*^, Fernando García^1^

##### ^1^Centro de Investigación en Enfermedades Tropicales y Facultad de Microbiología, Universidad de Costa Rica, Costa Rica

Correspondence: Jose Arturo Molina-Mora - jose.molinamora@ucr.ac.cr

*BMC Bioinformatics* 2020, **21(Suppl 20)**: O7

De novo genome assembly is an open challenge in bioinformatic analyzes. In order to select the reconstructed sequence that is closest to the real genome, different approaches to evaluate and select assemblies have been implemented. First, different metrics have been used that are related to the number and size of pieces obtained with respect to the expected sequence, the Contiguity. Other comparison strategies have focused on the ability to reconstruct essential genes and known elements, the Completeness (how much of the genome is represented by the pieces of the assembly). Also, accuracy between the sequenced and the expected bases has been a matter of discussion, which depends on DNA sequencing technologies. This can be referred as Correctness, how well those pieces accurately represent the genome sequenced.

In our previous work we conceptualized the criterion 3C (contiguity, completeness and correctness) as a set of metrics that can be used to benchmark genome assemblies. We assessed this criterion using the Costa Rican *Pseudomonas aeruginosa* AG1 isolate as model [1].

For the current study, two new clones of *P. aeruginosa* AG1 were obtained after culturing using high ciprofloxacin concentration media, in which this bacteria regularly do not grow. In comparison to the reference genome (*P. aeruginosa* PAO1), it was estimated that *P. aeruginosa* AG1 and the two new clones had ~ 1 Mb additional DNA sequence in its genome, justifying a de novo assembly. All genomes were sequenced using short- (Illumina) and long-reads (Nanopore) technologies. A benchmark of 10 approaches was done for each strain, considering different algorithms (assemblers) and DNA sequencing technologies for hybrid and non-hybrid models. The 3C criterion was used for each strain to select the best assembly.

From the benchmarking results a better performance of long reads technologies to solve repeated zones (impacting contiguity) and the fidelity (correctness) obtained by short reads technology stand out. Despite the fact that some assembly algorithms achieved a single contig as expected, surprisingly a large number of fragmented genes (frameshifts) were identified for long reads assemblers (affecting correctness and completeness). Thus, assessment using 3C criterion showed an improved performance for a hybrid assembly approach, with the best advantages of each sequencing technology.

This steps are critical not only to understand the genome architecture of these strains, but also for further studies at other—omic levels, as we recently published for the transcriptomic response to ciprofloxacin in *P. aeruginosa* AG1 [2].

**References**Molina-Mora, J.-A.; Campos-Sánchez, R.; Rodríguez, C.; Shi, L.; García, F. High quality 3C de novo assembly and annotation of a multidrug resistant ST-111 Pseudomonas aeruginosa genome: Benchmark of hybrid and non-hybrid assemblers. *Sci. Rep.* 2020, *10*, 1392, https://doi.org/10.1038/s41598-020-58319-6.Molina-Mora, J.A.; Chinchilla, D.; Chavarría, M.; Ulloa, A.; Campos-Sanchez, R.; Mora-Rodríguez, R.A.; Shi, L.; García, F. Transcriptomic determinants of the response of ST-111 Pseudomonas aeruginosa AG1 to ciprofloxacin identified by a top-down systems biology approach. *Sci. Rep.* 2020, *10*, 1–23, https://doi.org/10.1038/s41598-020-70581-2.

## O8

### Pannopi: prokaryotic genome assembly and annotation pipeline

#### Danil S. Zilov^1*^, Aleksey S. Komissarov^1^

##### Applied Genomics Laboratory, SCAMT Institute, ITMO University, Saint-Petersburg, 191,002, Russia

Correspondence: Danil S. Zilov - zilov@scamt-itmo.ru

*BMC Bioinformatics* 2020, **21(Suppl 20)**: O8

The emergence of a new generation of sequencing for the first time allowed us to significantly accelerate and reduce the cost of determining the complete sequence of millions of genomes of organisms, from bacteria to human. Scientists are now looking to miniaturize and automate the sequencing process, increase the amount of data obtained, and reduce the cost of it. It is clear from bioinformatics that as the cost of sequencing decreases, the number of data processed will increase. It is necessary to identify and automate the areas of analysis that are routine.

We created Pannopi—a scalable, easy-to-use assembly and annotation pipeline based on a hierarchical pan-genome graph. The program performs a large-scale analysis of the nucleic acid sequence of bacteria from preparation to functional annotation. The process runs from the preparation of sequence reads to genome assembly, through cleaning up the genome from external contamination to structural and functional annotation. Quality control is carried out throughout the process. Pannopi has tests and benchmarks not only for genome assembly, but also for its annotation using eight genomes from different taxonomic groups. This allows new annotation methods to be tested and benchmarking quickly.

Pipeline includes the most advanced and effective tools for genomic annotation and allows for flexible customization of their use. So that the user can select between a few genome assemblers and annotators or even to run all of the tools for subsequent compare. Also, Pannopi allows users to select the taxons for pan-genome comparative genomics and required modules; it can be used on a separate command-line program or through a web interface. Pannopi output includes includes: raw data quality control; assembly; cleaned from contamination assembly; assembly quality control; structural annotation; functional annotation; pangenome-based comparative annotation; lists of antibiotic resistance and virulence genes, plasmids, phages, IS elements, tandem repeats, mlst-type, and serotype.

## O9

### Assembly and Annotation of Ashkenazi reference genome

#### Aleksey V. Zimin^1,2,*†^, Alaina Shumate^1,2,†^, Rachel M. Sherman^1,3^, Daniela Puiu^1,3^, Justin M. Wagner^4^, Nathan D. Olson^4^, Mihaela Pertea^1,2^, Marc L. Salit^5^, Justin M. Zook^4^ and Steven L. Salzberg^1,2,3,6^

##### ^1^Center for Computational Biology, Johns Hopkins University, Baltimore, MD; ^2^Department of Biomedical Engineering, Johns Hopkins University, Baltimore, MD; ^3^Department of Computer Science, Johns Hopkins University, Baltimore, MD; ^4^National Institute of Standards and Technology, Gaithersburg, MD; ^5^Stanford University, Stanford, CA; ^6^Department of Biostatistics, Johns Hopkins University, Baltimore, MD

Correspondence: Aleksey V. Zimin - alekseyz@jhu.edu

*BMC Bioinformatics* 2020, **21(Suppl 20)**: O9

^†^These authors contributed equally to this work.

As the sequencing technologies advance and costs decrease, it is now becoming possible to produce individual reference genomes that rival the quality of the well-established references. In this project we produced new population-specific human reference genome. For the initial assembly we used publicly available Illumina, Oxford Nanopore ultralong and PacBio HiFi data for Human Genome Project HG002 individual from Ashkenazi jewish trio. These data are available from Genome In A Bottle (GIAB) project. We assembled the reads with MaSuRCA genome assembler version 3.3.1, and then used MaSuRCA chromosome scaffolder to validate, order and orient the assembled contigs based on their alignments to the human reference genome GRCh38.p12. GRCh38 reference contains a lot of repetitive and therefore hard-to-assemble regions some of which have been put together using labor-intensive manual curation. Even the long reads from Nanopore and PacBio are still unable to properly resolve many of these regions. Thus, rather than having gaps in the chromosome sequences, wherever possible, we filled them using GRCh38.p12 sequence in lowercase letters.

The new reference that we call Ash1, has more sequence placed on the chromosomes: 2,973,118,650 nucleotides as compared to 2,937,639,212 in GRCh38. While GRCh38 is a mosaic of many different individual genomes, our reference represents a single traditional haplotype-merged individual genome. We annotated the genome by transferring the CHESS 2.0 annotation from GRCh38.p12 reference using a novel Liftoff tool. The Ash1 annotation identified 20,157 protein-coding genes, of which 19,563 are > 99% identical to their counterparts on GRCh38.p12. 40 of the protein-coding genes present in GRCh38.p12 are missing from Ash1. However, all these genes are members of multi-gene families for which Ash1 contains other copies. We found no cases at all where a gene present in GRCh38.p12 totally missing from Ash1. Alignment of Illumina reads from an unrelated part-Ashkenazi (~ 70%) individual PGP17 from Personal Genome Project to Ash1 identified about 1 million fewer homozygous SNPs than alignment of those same sequences to the more-distant GRCh38 reference, illustrating one of the benefits of having a population-specific reference genome.

## O10

### Specialized Metabolism Gene Clusters from Red Sea Brine Pool Microbial Metagenomes

#### Laila Ziko^1,2*^, Mustafa Adel^1,2^, Mohamed N. Malash^2,3^ and Rania Siam^2^

##### ^1^Graduate Program of Biotechnology, School of Sciences and Engineering, American University in Cairo, New Cairo, Cairo 11835, Egypt; ^2^Biology Department, School of Sciences and Engineering, American University in Cairo, New Cairo, Cairo 11835, Egypt; ^3^Microbiology and Immunology Department, Faculty of Pharmacy, Ahram Canadian University, Giza 12581, Egypt

Correspondence: Laila Ziko - laila.adel@aucegypt.edu

*BMC Bioinformatics* 2020, **21(Suppl 20)**: O10

Mining for specialized metabolism gene clusters (SMGCs) is one approach to finding new antibacterial and anticancer natural products, especially from under-explored environments. Microbial metagenomes from Atlantis II Deep, Discovery Deep and Kebrit Deep Red Sea brine pools were shotgun sequenced and 2751 Red Sea brine SMGCs were detected. The Red Sea brine SMGCs were found to be potentially encoding for natural products pertaining to 28 classes, that were functionally grouped into three main categories, which comprise the following diverse chemistries -in addition to hybrid clusters: (1) saccharides, fatty acids, aryl polyenes, acyl-homoserine lactones, (2) terpenes, ribosomal peptides, non-ribosomal peptides, polyketides, phosphonates and (3) polyunsaturated fatty acids, ectoine, ladderane and others. We recently reported our findings, and here we will focus on the specific methodology of SMGCs detection in metagenomic samples, and on a particular selected group of natural products, which are the Ribosomally synthesized and post-translationally modified peptides (RiPPs). Although RiPPs constitute only 0.78% of the total Red Sea brine SMGCs, they are technically feasible to test in the lab, and thus it can be selected for prioritization for downstream experimentation. Moreover, several earlier studies have reported RiPPs belonging to similar classes, which exhibited antibacterial and/or anticancer effects. Bacteriocins (17 SMGCs), saccharide-bacteriocin hybrid clusters (3 SMGCs), Microcins (3 SMGCs) and Lanthipeptides (2 SMGCs), constitute the detected Red Sea brine RiPPs. In addition to our earlier reported results, here we will focus more on the methodology and recommendations for optimal mining microbial metagenomes for SMGCs, furthermore, we focus on and prioritize an additional selected group (RiPPs) for recommendation to the experimental work to validate and highlight the importance of the implemented methodology.

## O11

### Metagenomic analysis using k-mer-based tools reveal cyanobacteria and heavy metal response genes in a copper mining site in Benguet Province, Philippines

#### Libertine Rose S. Sanchez, Ernelea P. Cao

##### Institute of Biology, College of Science, University of the Philippines, Diliman, Quezon City, Philippines

Correspondence: Libertine Rose S. Sanchez - lssanchez@up.edu.ph

*BMC Bioinformatics* 2020, **21(Suppl 20)**: O11

Heavy metal contamination in mining sites causes growth inhibition of green vegetation. Fortunately, there are photosynthetic cyanobacteria that can survive in such environments. Surface water samples were collected from three sampling points in each Tailings Storage Facility (TSF) of the copper mining corporation, Philex mines in Benguet Province, Philippines such as the re-vegetated inactive Philex TSF1 and the currently active Philex TSF3. Genomic DNA was extracted from water samples and subjected to 2 × 150 paired-end shotgun sequencing. A total of 72.87 Gbases raw reads after a QC [1] were successfully assembled using St. Petersburg genome assembler (metaSPAdes) [2] and the quality was assessed with metaQUAST [3]. The default and custom-based approaches for both CLARK [4, 5] and Kraken2 [6] metagenomic classifiers were used in determining taxonomic assignments to contigs using k-mer matches. The default CLARK classified a large number of sequences across all sampling points. Their taxonomic assignments revealed the top five cyanobacteria, namely, the unicellular *Synechococcus*, *Cyanobium* and *Gloeobacter*, the filamentous, non-heterocystous *Leptoplyngbya*, and the heterocystous *Nostoc.* Custom-based CLARK identified *Leptolyngbya*, which is about 3–4% of the assembled contigs. On the other hand, Kraken2 uncovered the most dominant Rank Order Nostocales ranging from 0.05% to 0.63% of the classified sequences. The cyanobacterial custom-based Kraken2 showed a large number of sequences belonging to the filamentous *Fischerella* and *Trichodesmium* sp. in the re-vegetated TSF1. A unicellular *Microcystis* and filamentous *Nostoc*, *Spirulina* and *Pseudanabaena* dominated the active TSF3. CLARK was able to discriminate cyanobacteria up to the species level, while the default Kraken2 classifier was able to distinguish up to the dominant Rank Order taxon. Although the custom-based CLARK detected more cyanobacteria at the Rank Order level compared to Kraken2, the former was only able to determine a single cyanobacterium at the genus level. Kraken2 produced varying identifications of cyanobacteria in all sites, while CLARK consistently identified the same cyanobacterial species in all sites. Data sets were deposited at DDBJ/ENA/GenBank BioProject ID PRJNA504923 under the accession VFQP0000000000, VFQQ000000000, VFQR000000000, VFQS000000000, VFQT000000000 and VFQU0000000. Protein-coding sequences output from Prokka [7] that were evaluated using eggNOG [8] revealed genes conferring stress response to Cu^2+^, Zn^2+^, Pb^2+^, Cd^2+^, Ca^2+^ metal ions and *smt* metallothionein. These genes are reported to be responsible for the efflux/transport functions and heavy metal resistance that can be major attributes of cyanobacterial species for their survival to extreme metal conditions. This is the first report of cyanobacteria in a copper mining site in Benguet Province that were analyzed using shotgun metagenomics.

**References**Andrews S. FastQC: A quality control for high throughput sequence data. 2010. Available from: https://www.bioinformatics.babraham.ac.uk/projects/fastqc.Nurk S, Meleshko D, Korobeynikov A, Pevzner PA. metaSPADES: a new versatile metagenomic assembler. Genome Res 2017;27: 823–834. Available from: https://www.ncbi.nlm.nih.gov/pubmed/28298430Milkheenko A, Savaliev V, Girevich A. MetaQuast: evaluation of metagenome assemblies. Bioinformatics 2016;32(7):1088–1090. Available from: 10.1093/bioinformatics/btv697.Ounit R, Lonardi S. Higher classification sensitivity of short metagenomic reads with CLARK-S. Bioinformatics 2016;32(24): 3823–3825. Available from: 10.1093/bioinformatics/btw542.Ounit R, Wanamaker S, Close Tj, Lonardi S. CLARK: fast and accurate classification of metagenomic and genomic sequences using discriminative k-mers. BMC Genomics 2014;16: 236. Available from: 10.1186/s12864-015-1419-2.Wood DE, Salzberg SL. Kraken: ultrafast metagenomic sequence classification using exact alignments. Genome Biol 2014;14: R46. Available from: https://ccb.jhu.edu/software/kraken/Seemann, T. Prokka: rapid, prokaryotic genome annotation. Bioinformatics 2014;30(14): 2068–2069. Available from: 10.1093/bioinformatics/btu153.Huerta-Cepas J, Forslund K, Cuelho Lp, Szklarczyk D, Jensen Lj, Von Mering C, Bork P. Fast genome-wide functional annotation through orthology assignment by eggNOG-maper. Mol Biol Evol 2017;34(8): 2115–2122. Available from: 10.1093/molbev/msx148.

## O12

### The search for genetic risk factors of ischemic stroke with the genome-wide association study and machine learning methods

#### Gennady Khvorykh^1,*^, Margarita Rogacheva^2^, Ruslan Sharypov^3^, Andrey Khrunin^1^, Aleksandr Dyakonov^3^, Svetlana Limborska^1^, Alexei Fedorov^1,4^

##### ^1^Department of Molecular Bases of Human Genetics, Institute of Molecular Genetics of National Research Centre «Kurchatov Institute», Moscow, 123182, Russia; ^2^Faculty of Biology, Saint-Petersburg State University, Saint-Petersburg, 199034, Russia; ^3^Faculty of Computational Mathematics and Cybernetics, Lomonosov Moscow Statue University, Moscow, 119991, Russia; ^4^Department of Medicine, The University of Toledo, Toledo, OH, 43614-2598, USA

Correspondence: Gennady Khvorykh - khvorykh@img.ras.ru

*BMC Bioinformatics* 2020, **21(Suppl 20)**: O12

The ischemic stroke (IS) is a neurological deficit of sudden onset due to brain infarction. It is the primary cause of acquired disability in adults and a leading cause of death. The disease is multifactorial where genetic factors have a certain contribution. Numerous genetic polymorphisms are believed to increase the risk of IS. The advent of genome-wide genotyping caused a wave of genome-wide association studies (GWAS). At least ten candidate-genes associated with IS were previously described. Here we present the first round findings of single nucleotide polymorphisms (SNPs) associated with the development of IS in individuals of the Eastern Slavic ancestry. The results were obtained with GWAS and machine learning (ML) approaches.

The case and control groups consisted of 1051 and 421 individuals, correspondingly. They were genotyped with several types of DNA-microarrays. Upon meeting the requirement of quality control, we obtained about 82,000 autosomal SNPs in the merged dataset. The GWAS included a conventional chi-square test, an exact Fisher test and the Bayes factor method. Two SNPs were found to be significant at least by two methods: rs7699716 (chr4: 96648015, OR 2.41, p-value 2.35e-11) and rs7796855 (chr7: 49627992, OR 1.52, p-value 3.7e-07).

The ML approaches involved Support Vector Machine, k-Nearest Neighbors, Random Forest, Logistic Regression (LR), Gradient Boosting, and Neural Network (NN). The binary classifiers were fitted with different dimension reduction techniques (Singular Vector Decomposition, Diet Networks, and Uniform manifold approximation). The highest accuracy (ROC-AUC) of 0.697 was achieved with the NN method. The effect of SNP on the outcome variable was estimated with SHAP values for LR model. The top ranked SNP rs7699716 coincided with the GWAS results.

This SNP is located in the first intron of the *UNC5C* gene coding a netrin receptor. Netrins direct axon extension and cell migration during neural development. *UNC5C* is known to be associated to Alzheimer disease. The SNP rs7796855 is intergenic. It has been associated with Parkinson disease. The results obtained suggest that IS and the neurogenerative diseases have common basic mechanisms for developing of brain injuries.

During the second round of the research, which will involve larger groups of samples, we plan to extend the genotypic data to verify the findings made.

The study was funded by RFBR (Russian Foundation for Basic Research) according to the research project No 19-29-01151.

## O13

### Efficient dynamic associative dictionary for large k-mer sets

#### Yoann Dufresne*^1^, Camille Marchet^2^, Rayan Chikhi^1^, Antoine Limasset^2^

##### ^1^Department of Computational Biology, C3BI USR 3756 CNRS, Institut Pasteur, Paris, France; ^2^ Univ. Lille, CNRS, UMR 9189—CRIStAL, F-59000 Lille

Correspondence: Yoann Dufresne - yoann.dufresne0@gmail.com

*BMC Bioinformatics* 2020, **21(Suppl 20)**: O13

**Motivation** Since BLAST introduced the seed and extend paradigm, indexing fixed-length words (*k*-mers) from a set of sequences is the bread and butter of most algorithms and methods relying on sequence similarity. Due to the ever-increasing amount of available reference genomes, there is a growing interest in global approaches able to take into account a very broad sequence range. Ambitious applications such as pangenomics or metagenomics require to index billions of distinct *k*-mers and would benefit from incorporating as many reference genomes as possible. Recently, the problem of representing massive *k*-mer sets with low memory usage and a high throughput caught the community’s interest. In the last few years, several efficient methods (Pufferfish [1], Bifrost [2], BLight [3], REINDEER [4], Kallisto [5], Jellyfish [6], SRC [7]) were proposed with various applications: *k*-mer counting, quantification, assembly, …

Some implementations are specific to their main application, others are generic libraries that can fit various purposes. Jellyfish indexes *k*-mers using an efficient lock-free dynamic hash table scheme to enable fast *k*-mer counting. Such a scheme needs to store each *k*-mer in memory, which represents a memory cost of several bytes per *k*-mer (4 bytes for 31-mers). Probabilistic dictionaries [7] can use less than 2 bytes per *k*-mer at the expense of a low false-positive rate. Recent improvements provided efficient deterministic *k*-mer set representations, exploiting nucleotide redundancy in *k*-mer sets to lower the memory cost [1], and *k*-mer partitioning to further reduce the storage cost and raise cache coherency [3]. However, the efficiency of some of those methods relies on their static aspect. Large construction or update costs make them unfit to some applications where insertions or deletion are required. For instance, the rapid acquisition of new data for microbial pangenomes could benefit from dynamic structures. Large scale dynamic de Bruijn graphs [2, 8] are another possible application that is gaining traction.

**Results** We present BRISK (Brisk Reduced Index for sequence of *k*-mers) a resource-efficient dynamic dictionary able to associate value to *k*-mers without false positives. It relies on three main ideas.

First, instead of storing *k*-mers independently, we store super-*k*-mers, a sequence of *k*-mers that share the same minimizer, to reduce the amount of nucleotides required to encode overlapping *k*-mers. We partition super-*k*-mers according to their minimizers, which allows us to work on smaller structures, and improves cache coherence. Second, we represent a partition as a sorted list of super-*k*-mers to ensure fast retrieval of *k*-mers. Lastly, we use less nucleotides by encoding only the suffix and the prefix of a super-*k*-mer without its minimizer. In practice using this scheme, we can encode on average [9] eight 31-mers into a single super-*k*-mer that can fit on a 64 bits integer. The larger the minimizer size, the faster the queries but also the larger the space overhead. That means that queries can be adapted for different space/time tradeoffs. Furthermore, index usage is highly cache-coherent as querying several *k*-mers sharing the same minimizer only requires one random memory access.

## References


Almodaresi, F., Sarkar, H., Srivastava, A. and Patro, R., 2018. A space and time-efficient index for the compacted colored de Bruijn graph. *Bioinformatics*, *34*(13), pp.i169–i177.Holley, G. and Melsted, P., 2019. Bifrost–Highly parallel construction and indexing of colored and compacted de Bruijn graphs. *BioRxiv*, p.695338.Marchet, C., Kerbiriou, M. and Limasset, A., 2019, April. Indexing De Bruijn graphs with minimizers. In *Recomb seq*.Marchet, C., Iqbal, Z., Gautheret, D., Salson, M. and Chikhi, R., 2020. REINDEER: efficient indexing of *k*-mer presence and abundance in sequencing datasets. *ISMB.*Bray, N.L., Pimentel, H., Melsted, P. and Pachter, L., 2016. Near-optimal probabilistic RNA-seq quantification. *Nature biotechnology*, *34*(5), pp.525–527.Marçais, G. and Kingsford, C., 2011. A fast, lock-free approach for efficient parallel counting of occurrences of *k*-mers. *Bioinformatics*, *27*(6), pp.764–770.Marchet, C., Lecompte, L., Limasset, A., Bittner, L. and Peterlongo, P., 2020. A resource-frugal probabilistic dictionary and applications in bioinformatics. *Discrete Applied Mathematics*, *274*, pp.92–102.Crawford, V. G., Kuhnle, A., Boucher, C., Chikhi, R., & Gagie, T., 2018. Practical dynamic de Bruijn graphs. *Bioinformatics,* 34(24), pp.4189–4195.Baharav, T.Z., Kamath, G.M., David, N.T. and Shomorony, I., 2020, May. Spectral Jaccard Similarity: A new approach to estimating pairwise sequence alignments. In *International Conference on Research in Computational Molecular Biology* (pp. 223–225). Springer, Cham.

## P1

### Secondary structure prediction by combination of formal grammars and neural networks

#### Semyon Grigorev^1,2*^, Dmitry Kutlenkov^1,2^, Polina Lunina^1,2^

##### ^1^Saint Petersburg State University, St. Petersburg, 199034, Russia; ^2^JetBrains Research, St. Petersburg, 197374, Russia

Correspondence: Semyon Grigorev - semyon.grigorev@jetbrains.com

*BMC Bioinformatics* 2020, **21(Suppl 20)**: P1

Secondary structure is known to have a crucial impact on RNA molecules functioning, therefore, development of algorithms for secondary structure modeling and prediction is a fundamental task in computational genomics. Among other methods, secondary structure can be theoretically described by means of formal grammars [1, 2].

An approach for sequences secondary structure analysis by combination of formal grammars and neural networks was proposed in [3, 4]. In this work, we apply this approach to RNA secondary structure prediction. Secondary structure can be described as composition of stems having different heights and loop sizes [5]. We use context-free grammar from [3] to encode the most common kinds of stems and parsing algorithm [6] to find such stems in sequences. Note that this grammar describes only the classical base pairs and cannot express pseudoknots. The result of a matrix-based parsing algorithm for some sequence is a boolean matrix that represents all the theoretically possible stems in terms of grammar, but the real secondary structure cannot contain all of them at once and, besides, there can be more complex, not expressible in given grammar elements. Therefore, parsing matrices require further processing and we propose to use a neural network to handle them in order to generate an actual secondary structure.

For experimental research we took sequences from RnaCentral [7] database and as reference data for network training we used the output of CentroidFold tool [8]: contact matrices that represent connections between nucleotides in secondary structure. We transformed parsing matrices and contact maps to black-and-white images. These images were used for training the generative neural network which takes a parsing-provided image as an input and transforms it to the maximal approximation of the considered contact map. We applied deep residual networks with the local alignment algorithm at the end of the sequence of layers.

We trained models with and without alignment on several datasets with fixed sequence length interval and estimated them by precision, recall and F1 score metrics calculated for numbers of correctly and incorrectly guessed contacts for each image. All models showed F1 score up to 70% and we discovered that the smaller the window size, the more accurate the model, moreover, alignment significantly improves precision of neural networks due to removing the contacts that break the secondary structure.

To conclude, the set of experiments confirmed that the proposed approach is applicable to secondary structure prediction problem and further research is required.

**Acknowledgments**

The research was supported by the Russian Science Foundation Grant 18-11-00100 and a grant from JetBrains Research.

**References**Dowell RD, Eddy SR. Evaluation of several lightweight stochastic context-free grammars for RNA secondary structure prediction. BMC Bioinformatics. 2004;5(1):71.Knudsen B, Hein J. RNA secondary structure prediction using stochastic context-free grammars and evolutionary history. Bioinformatics. 1999 Jun 1;15(6):446–54.Grigorev S, Lunina P. The composition of dense neural networks and formal grammars for secondary structure analysis. In De Maria E, Gamboa H, Fred A, editors, BIOINFORMATICS 2019—10th International Conference on Bioinformatics Models, Methods and Algorithms, Proceedings; Part of 12th International Joint Conference on Biomedical Engineering Systems and Technologies, BIOSTEC 2019. SciTePress. 2019. p. 234–241Grigorev S, Lunina P. Improved Architecture of Artificial Neural Network for Secondary Structure Analysis. BMC Bioinformatics. 2019;20(S17). P2.Quadrini M, Merelli E, Piergallini R. Loop Grammars to Identify RNA Structural Patterns. In: Proceedings of the 12th International Joint Conference on Biomedical Engineering Systems and Technologies [Internet]. SCITEPRESS—Science and Technology Publications; 2019.Azimov R, Grigorev S. Context-free path querying by matrix multiplication. In Bhattacharya A, Fletcher G, Roy S, Arora A, Larriba Pey JL, West R, editors, Proceedings of the 1st ACM SIGMOD Joint International Workshop on Graph Data Management Experiences and Systems (GRADES) and Network Data Analytics (NDA), GRADES-NDA 2018. Association for Computing Machinery. 2018. a5. (Proceedings of the 1st ACM SIGMOD Joint International Workshop on Graph Data Management Experiences and Systems (GRADES) and Network Data Analytics (NDA), GRADES-NDA 2018).Sweeney BA, Petrov AI, Burkov B, Finn RD, Bateman A, Szymanski M, et al. RNAcentral: a hub of information for non-coding RNA sequences. Nucleic Acids Research. 2019 Jan 8;47(D1):D221-D229.Hamada M, Kiryu H, Sato K, Mituyama T, Asai K. Prediction of RNA secondary structure using generalized centroid estimators. Bioinformatics. 2009 Feb 15;25(4):465–73.

## P2

### Dissecting the evolutionary mechanisms of the 3-domain Cry toxins diversity

#### Anton E. Shikov^1,2*^, Yury V. Malovichko^1,2^, Anton A. Nizhnikov^1,2^, Kirill S. Antonets^1,2^

##### ^1^All-Russia Research Institute for Agricultural Microbiology (ARRIAM), Pushkin, St. Petersburg, Russia; ^2^ St. Petersburg State University, St. Petersburg, Russia

Correspondence: Anton E. Shikov - a.shikov@arriam.ru; Kirill S. Antonets - k.antonets@arriam.ru

*BMC Bioinformatics* 2020, **21(Suppl 20)**: P2

Biologicals based on the entomopathogenic gram-positive bacteria, *Bacillus thuringiensis*, represent one of the most widespread biopesticides. The potency and specificity of insecticidal action are determined mostly by insecticidal moieties produced mainly as the crystalline inclusions during the sporulation growth phase of the bacterium. Although diverse virulence factors are produced by *B. thuringiensis*, Cry toxins refer to the most useful and agriculturally applicable biopesticides. Cry toxins and their subset, 3-D (three-domain) Cry toxins, exhibit a wide range of affected hosts and high specificity. Unfortunately, an emerging resistance of insects to these toxins due to mutations in hosts' receptors retards efficient pest management. The two strategies that could contribute to solving this issue are the search for novel toxins and the construction of artificial toxins through the domain shuffling. To discover new 3-D Cry toxins in genomic data, we have recently developed an HMM-based tool called CryProcessor that retrieves sequences of 3-D Cry toxins from large datasets and provides an opportunity to get the layout of individual domains [1]. This tool outperforms its analogs in terms of accuracy, speed, and throughput. Cry toxins' domain layout provided by CryProcessor could facilitate the development of chimeric toxins by accelerating in silico construction of chimeric toxins. Considering the diversity of Cry toxins, one generally accepted yet not lucidly validated hypothesis links this diversification of the 3-D Cry toxins with domains' exchanges between them. To fulfill this gap, here we conducted a large-scale phylogenetic study of the 3-D Cry toxins. Using CryProcessor, we screened the IPG and Genbank databases and identified 600 novel toxins, which were then merged with toxins from the *Bt* Nomenclature. We constructed phylogenetic trees based both on full sequences and separate domains. The evaluation of topological differences between the trees revealed a dissimilarity between the topology of the full sequence-based tree and the domain-only trees. We then screened sequences for signals of recombination events. As a result, we revealed 50 recombination events that belonged to each of the domains. Our results indicate that recombination events represent a pivotal mechanism for the evolution and diversification of 3-D Cry toxins. A more in-depth look into the history of recombination events would allow us to understand evolutionary mechanisms originating the Cry toxins diversity, and to develop new toxins precisely and efficiently.

This study was supported by the Russian Science Foundation (20-76-10044).

**Reference**Shikov A.E., Malovichko Yu.V., Skitchenko R.K., Nizhnikov A.A., Antonets K.S. No more tears: mining Sequencing data for novel *Bt* Cry toxins with CryProcessor. Toxins. 2020; 12(3): 204

## P3

### Genome assembly of heterozygous tropical trees—will the real (pan)genome stand up?

#### Pumipat Tongyoo^1,2^, Adisorn Chaibang^3^, Hugo A. Volkaert^1,2*^

##### ^1^Center for Agricultural Biotechnology, Kasetsart University Kamphaengsaen Campus, Nakhon Pathom 73140, Thailand; ^2^Center of Excellence on Agricultural Biotechnology AG-BIO/PERDO-CHE, Ministry of Education, Bangkok 10900, Thailand; ^3^Department of Science, Faculty of Liberal Arts and Science, Kasetsart University Kamphaengsaen Campus, Nakhon Pathom 73140, Thailand

Correspondence: Hugo A. Volkaert - kpshgv@ku.th; hugo.a.volkaert@gmail.com

*BMC Bioinformatics* 2020, **21(Suppl 20)**: P3

We study the genetic diversity in trees of the deciduous forests in Thailand to understand their adaptation to past environments and predict their response to future climate changes. High-throughput sequencing greatly enhances population genetics studies through its power to genotype individuals at multiple loci. Though methods exist for obtaining genotypes and polymorphism data without a reference genome, having a reference sequence at least for the single-copy regions of the genome does help when one would like to compare diversity parameters across populations and species.

Through k-mer frequency analysis at multiple k-mer lengths the tree genomes (*Xylia xylocarpa*, *Dipterocarpus tuberculatus*, *Gluta usitata*, *Dalbergia* spp., *Afzelia xylocarpa*) are shown to be highly heterozygous. The k-mer length at which the peak frequency of homozygous k-mers equals the peak frequency of heterozygous k-mers is proposed as a reliable measure to compare the level of polymorphism across heterozygous species.

As the individual genomes are highly heterozygous, assembly programmes struggle to deliver an acceptable reference sequence even for the non-repetitive part of the genome. Platanus, Platanus-allee, SPAdes and Meraculous were compared for their genome assembly capabilities. None of the programmes delivered a usable reference genome from the sequence data, approximately 12–25 × genome coverage of a single library sequenced by Illumina® paired-end reads of 101 or 150 nucleotides. Assemblies are highly fragmented, generally producing a single contig in short regions of lower heterozygosity and two contigs for regions where the haplotypes are highly divergent with breaks in between.

We are developing a “haplotype-specific k-mer walking” assembly pipeline which is based on identifying trusted single-copy k-mers and extending the two chromosomal copies concurrently using read pairs containing those k-mers exactly. Through this pipeline contigs longer than 10,000 bp can be assembled spanning multiple scaffolds produced by regular genome assemblers. Moreover, a sufficient number of read pairs contain two polymorphisms allowing not only genome assembly but also phasing of polymorphic sites at the same time. The k-mer selection and walking process needs to be further automated and parallelized.

The data from moderate to low coverage Illumina^®^ genome sequencing contain sufficient information for the assembly of long contigs representing both haplotypes derived from the two genomes in heterozygous individuals.

## P4

### Metagenomic analysis of the soil microbiota associated with plant gigantism of the unique Siberian Chernevaya Taiga

#### Mikhail Rayko^1*^, Anastasia Kulemzina^2*^, Evgeny Abakumov^3^, Georgy Istigechev^4^, Evgeny Andronov^5,6^, Nikolay Lashchinsky^7^, Alla Lapidus^1,#^

##### ^1^Center for Algorithmic Biotechnology, St. Petersburg State University, St. Petersburg, 990034, Russia; ^2^Institute of Molecular and Cellular Biology SB RAS, Novosibirsk, 630090, Russia; ^3^Department of Applied Ecology, Saint Petersburg State University, St. Petersburg, 99034, Russia; ^4^BIO-GEO-CLIM Laboratory, Tomsk State University, Tomsk, 634050, Russia; ^5^All-Russian Research Institute for Agricultural Microbiology, St. Petersburg, 196608, Russia; ^6^St. Petersburg State University, St. Petersburg, 1990034, Russia; ^7^Central Siberian Botanical Garden, Novosibirsk, 630090, Russia

^*^Equally participating authors.

Correspondence: Alla Lapidus - a.lapidus@spbu.ru

*BMC Bioinformatics* 2020, **21(Suppl 20)**: P4

Chernevaya taiga can be described as a boreal forest formation, limited in its spread by hyper humid sections of the Altai-Sayan mountainous region. It is characterized by a series of unique ecological traits, the most notable of which is the gigantism of the perennial grassy plants and bushes.

The main goal of the study is to discover and parametrize the main factors that affect the anomalously elevated effective fertility of the Chernevaya taiga soils, with the focus on the microbial communities. We aim to establish a link between the distinct properties of Chernevaya taiga with the chemical parameters of the soil, the rate of moisturization, the unique composition of the microbiota and/or the aggregate of all of these factors.

Based on 16S analysis of the soils from two Chernevaya taiga locations (Novosibirsk and Tomsk regions) and control soils, we found that the richness of the soil microbiota is decreasing significantly with increasing sampling depth. The taxonomic structure of the microbiota of the top layers (0–15 cm) has similar properties in the different geographical locations of the Chernevaya taiga. The most prevalent phyla in the top layers of the Chernevaya taiga soils are Proteobacteria, Acidobacteria and Verrucomicrobia.

Differences in microbiota composition of the rhizosphere of *Crepis sibirica* between Chernevaya taiga and control regions were investigated using linear discriminant analysis effect size approach. We found bacterial taxa that are relatively abundant between both groups. Bacteroidetes (in particular Sphingobacteria and Cytophagia) were more abundant in the control group, and Actinobacteria (mostly Thermoleophilia) and Verrucomicrobia (Chthoniobacterales) in the Chernevaya taiga samples. It may indicate the specificity of the Chernevaya taiga microbiome, and its importance for the features of this biotope.

The reported study was funded by Russian Scientific Foundation (grant ID 19-16-00049).

## P5

### Plant virome analysis in search for new viruses: experience from CRI Prague, Czech Republic

#### Petr Komínek*, Marcela Komínková

Plant Virology and Phytoplasmatology Research Group, Crop Research Institute, 16106 Prague, Czech Republic

Correspondence: Petr Komínek - kominek@vurv.cz

*BMC Bioinformatics* 2020, **21(Suppl 20)**: P5

**Background** Viromes of fruit trees, vegetables, grapevines, and ornamentals were studied using high-throughput sequencing.

**Materials and methods** Total RNA was isolated from plant leaves, ribosomal RNA was removed and libraries for Illumina technology were prepared. Sequencing was done in MiSeq and NovaSeq machines, always for 2 × 150 nt.

Data were processed using Geneious and CLC Genomics Workbench, pipeline included removing duplicated reads, de novo assembly, contigs search by TBLASTX for plant viruses using a local database. Reads were then mapped back to contigs, coverage was calculated. In the case of novel viruses, mapped reads were checked by BLAST. The alignment of virus sequences with relative viruses was done using MUSCLE. Best fit nucleotide and aminoacid substitution models for each alignment and subsequent phylogenetic analysis was done by MEGA7 [1].

**Results** A number of viruses were identified in analyzed samples: six viruses and two viroids in grapevines; two viruses in fruit trees; 11 viruses including four novel viruses in ornamentals.

A novel member of the genus *Potyvirus*, family *Potyviridae* (single-stranded positive RNA genome), named *Pleione flower breaking virus*, was described infecting *Pleione* orchids [2].

A novel member of the family *Kitaviridae* (single-stranded positive RNA genome) was found in orchids *Vanilla* and *Eria*, tentatively named Vanilla-associated kitavirus. Phylogenetic analysis confirmed its belonging to the family *Kitaviridae*. The virus branched separately from recognized genera of the family, its designation into a genus is not certain.

A novel member of the family *Phenuiviridae* (single-stranded negative RNA genome) was found in orchids *Neotinea*, tentative name Neotinea-associated coguvirus.

A novel member of the family *Chrysoviridae* (double-stranded RNA genome) was found in orchids *Restrepia*, tentative name Restrepia-associated alphachrysovirus.

**Conclusions** Intuitive tools for analysis of high-throughput sequencing data like Geneious and CLC Genomics Workbench were successfully used to process large datasets obtained from Illumina sequencers. Genomes of four novel viruses from ornamental plants were assembled and annotated.

**Funding**

The work was supported by institutional support MZE-RO0418 from the Ministry of Agriculture of the Czech Republic.

**References**Kumar S, Stecher G, Tamura K. MEGA7: Molecular Evolutionary Genetics Analysis version 7.0 for bigger datasets. Mol Biol Evol. 2016; 33:1870–1874.Komínek P, Massart S, Pham K, van Leeuwen P, Komínková M. Characterisation of a novel virus infecting orchids of the genus Pleione. Virus Res. 2019; 261:56–59.

## P6

### Microbiome in tundra and forest tundra permafrost soils, southern Yamal, Russia

#### Ivan Alekseev^1*^, Aleksei Zverev ^1,2^, Evgeny Abakumov^1^

##### ^1^Saint Petersburg State University, Faculty of Biology, Department of Applied Ecology, Saint Petersburg, Russia; ^2^All-Russian Research Institute for Agricultural Microbiology, Saint Petersburg, Russia

Correspondence: Ivan Alekseev - alekseevivan95@gmail.com

*BMC Bioinformatics* 2020, **21(Suppl 20)**: P6

**Background** The use of metagenomic approaches for assessment of soil microbiome is highly promising, since it allows to identify the taxonomic markers and microbiological drivers of pedogenesis, which is especially relevant in regard of Arctic warming and its potential environmental risks. Despite very harsh climatic conditions, the diversity of soil bacterial communities of the Arctic is as high as in other biomes and still underestimated. This work is aimed at the assessment of microbial communities as well as identifying the main environmental factors affecting their structure and diversity in permafrost soils of Yamal peninsula, Russian Arctic.

**Materials and methods** DNA isolation was performed using the PowerSoil® DNA Isolation Kit (Mobio Laboratories, Solana Beach, CA, USA). The purified DNA templates were amplified with universal multiplex primers F515 5′—GTGCCAGCMGCCGCGGTAA-3′ and R806 5′—GGACTACVSGGGTATCTAAT-3′ [1] targeting the variable region V4 of bacterial and archaeal 16S rRNA genes. Amplicon libraries sequencing was performed by ILLUMINA MiSeq. Sequence data processing was carried out using “Trimmomatic” [2] and “QIIME” [3]. The main soil parameters were determined by standard procedures. Extraction of humic and fulvic acids was performed according to the method suggested by International Humic Substances Society.

**Results** The taxonomic analysis revealed the predominant microbial taxa in soil profiles of permafrost-affected soils in both tundra and forest tundra. Proteobacteria plylum has been previously found in polar soils and their habitats varied significantly in regards to changing nutrients and water availability, temperature regime and soil properties. Together with the presence of essential portions of organic remnants in the topsoil layers, quite warm and humid environments of southern Yamal explains the abundance of Acidobacteria phylum. Analysis of humus type in studied soils revealed the predominance of low-molecular fragments which testifies high mineralizing risks in system of humus substances in conditions of Arctic warming.

**Conclusions** Our results provide further evidence of high vulnerability and sensitivity of permafrost-affected soils organic matter to Arctic warming. pH range and nitrogen accumulation were found as the main environmental factors describing the microbial community diversity and composition in studied soils.

**Acknowledgements**

This work is supported by RSF grant № 17-16-01030 (sequencing), RFBR grant № 19-416-890002 (field work).

**References**Bates ST, Berg-Lyons D, Caporaso JG, Walters WA, Knight R, and Fierer N. Examining the global distribution of dominant archaeal populations in soil. ISME J. 2010; 5: 908–917.Bolger AM, Lohse M, Usadel B. Trimmomatic: A Flexible Trimmer for Illumina Sequence Data. Bioinformatics. 2014; 30: 2114–2120.Caporaso JG, Kuczynski J, Stombaugh J, et al. QIIME allows analysis of high-throughput community sequencing data. Nature methods. 2010; 7(5): 335–336.

## P7

### Metagenomic analysis as a bioinformatics tool to reconstruct the geochemical methane-driving processes in bottom sediments of the Yenisei River

#### Svetlana Evgrafova^1,3^*, Alexey Zverev^2^, Evgeny Abakumov^2^, Anna Detsura^3^, Anatoly Prokushkin^1,3^

##### ^1^V.N. Sukachev Institute of Forest *FRC KSC SB RAS, Krasnoyarsk, Russia;*^2^Saint Petersburg State University, Department of Applied Ecology, Saint Petersburg, Russia; ^3^Siberian Federal University, Krasnoyarsk, Russia

Correspondence: Svetlana Evgrafova - esj@yandex.ru

*BMC Bioinformatics* 2020, **21(Suppl 20)**: P7

New knowledge about methanogenic and methanotrophic Archaea diversity and activity in bottom sediments is important for understanding of processes involved to the transportation of carbon and biogenic compounds from terrestrial ecosystems to aquatic ones, and their biogeochemical transformation. From the other hand, the integration of functional diversity measurements into environmental microbiology research remains incomplete. The bottom sediments of lakes and rivers are the hotspots of methane production, but composition and activity of methanogenic and methanotrophic microorganisms remains poorly described for the bottom sediments of the great Arctic rivers. We aimed to use metagenomic analysis as a bioinformatics tool to reconstruct the geochemical methane-driving processes in bottom sediments of the Yenisei River.

We investigated the bottom sediments collected from 18 sites located between 56.0ºN and 67.4ºN along the Yenisei River. We used v4-region of 16 s rDNA gene sequencing data for assessing of microbiome composition from which we analyzed prokaryotes belonging to Achaea as well as methanotrofs. Sequences was obtained via Illumina MiSEQ, processing was performed in R. For trimming, filtering and ASVs determination followed by merging reads, standard pipeline for Dada2 was used. Phylogenetic tree was constructed by MAFFT and FastTree implementation in Qiime2. Rarefaction by minimum number (12833) reads, bargraphs, alpha- and beta-diversity metrics calculation was performed using phyloseq (vizualization by ggplot2).

In parallel with sediment sampling we collected samples of dissolved greenhouse gases (CO_2_ and CH_4_) by head space method and further measured CO_2_ and CH_4_ concentrations and δ13C-CH_4_ and δ13C-CO_2_. Metabolic pathways of methane production we revealed using δ13C-CH_4_ signature in anoxic incubation experiment with sediments.

As a result, we analyzed 52 bacterial and archaeal phyla, among which metanogenic Archaea constituted only 0.5–0.6% of sequences in the amplicon libraries (depending on sample). Methanogenic community structure of bottom sediments of the Yenisei River dominated by archaeons belonging to *Methanosarcina*, *Methanosaeta* and *Methanoregula*. The OTU abundance of these archaeons was larger in sediments collected between 56ºN and 61ºN. Along this channel segment the values of δ13C-CH_4_ in the dissolved methane has increased from -54 to -43‰ VPDB that indicated methylotrophic and acetoclastic methanogenesis. In the segment between 61ºN and 64ºN the OTU abundance of methanogenic Archaea decreased dramatically (5–190 times) which was accompanied by the sharp depletion of δ13C-CH_4_ up to − 60 to − 80 ‰ VPDB indicating the shift to hydrogenotrophic metabolic pathway of methane production. Also in this river area we observed increasing OTU abundance of anaerobic methanotrophs belonging to Candidatus Methanoperedens. Further North (64–67ºN) we observed enrichment of δ13C-CH_4_ and increasing in methanogenic community archaeons belonging to *Methanosarcina* and *Methanoregula*.

We think that NGS sequencing data can clarify taxonomical composition of metanotrophic and metanogenic communities of sediments, their activity and impact on geochemical methane-driving processes, and reveal active participants in those communities.

**Acknowledgements**

This study was supported by the grant from the RFBR, the project № 18-05-60203_Arctica and 19-05-50107.

## P8

### Installing and searching BLAST databases in a data science framework

#### Graham Alvare^1^, Abiel Roche-Lima^2^, Brian Fristensky^3*^

##### ^1^Access Norwest Co-op Community Health, Winnipeg, Canada; ^2^RCMI Program, Medical Science Campus, University of Puerto Rico, Puerto Rico; ^3^Department of Plant Science, University of Manitoba, Winnipeg, Canada

Correspondence: Brian Fristensky - brian.fristensky@umanitoba.ca

*BMC Bioinformatics* 2020, **21(Suppl 20)**: P8

Data science embodies a pipeline of processes: acquisition, cleaning and organization of data, quality control and assurance, validation, and downstream visualization and analytics. Because of the overwhelming number of tools for each of these steps, the challenge is often making them work in concert to facilitate a thorough and insightful analysis.

BIRCH [1] is a framework consisting of hundreds of bioinformatics tools, unified through the BioLegato family of programmable graphical applications [2]. Each BioLegato application instantiates a specific class of biological objects, packaging together the data and the methods for each object. We describe BioLegato applications for BLAST searches, implementing data science principles. For example, in **blncbi** the user retrieves sequences from NCBI using an Entrez query builder. Amino acid sequences matching the query pop up in **blprotein**, a BioLegato application for running protein-specific tasks. A protein can be selected for a BLAST search, and output will appear in **bpfetch,** a BioLegato spreadsheet object for protein hits. **blpfetch** makes it easy to scan hundreds of hits, refining the list into one or more subsets for retrieval. Sequences are retrieved to a new **blprotein** object for downstream analysis. For example, proteins aligned using mafft would pop up in a **blpalign** object. Compared to web applications, which show only a single step in a full-screen window, BioLegato objects can be arrayed on the screen to give a more global view of the pipeline.

BioLegato simplifies experimentation with the data at every step. Because output of each step appears in a new BioLegato object, there are no dead ends. Output from one step can be used directly as input for subsequent steps because BioLegato automates otherwise tedious and error-prone tasks like file format conversion. We call this process ad hoc pipelining. Ad hoc pipelining enables the user to learn from each step before going to the next. We also describe **blastdbkit**, a Python script run from BioLegato, for downloading and managing BLAST databases on the users's computer.

Together, BioLegato applications provide a seamless point and click pipeline for sequence database searches, within the context of the larger BIRCH system. New programs can be added to any BioLegato application by creating a file using BioLegato's PCD language [2], which specifies parameters to be set and a shell command to run the program. In this way, the core BIRCH functions can be integrated with locally-installed bioinformatics software.

BIRCH Web site: https://home.cc.umanitoba.ca/~psgendbFristensky B. BIRCH: A user-oriented, locally-customizable, bioinformatics system. BMC Bioinformatics. 2007; 8:5.Alvare GGM, Roche-Lima A, Fristensky B BioPCD—A Language for GUI Development Requiring a Minimal Skill Set. Int. J. Computer Appl. 2012; 57:9–16.

## P9

### Bioinformatics analysis of short-chain fatty acid production potential in the human gut microbiome

#### Maria S. Frolova^1^, Stanislav N. Iablokov^2,3^, Dmitry A. Rodionov^2, 4*^

##### ^1^Institute of Cell Biophysics, Russian Academy of Sciences, Pushchino, Russia; ^2^A.A. Kharkevich Institute for Information Transmission Problems, Russian Academy of Sciences, Moscow, Russia; ^3^P.G. Demidov Yaroslavl State University, Yaroslavl, Russia; ^4^Sanford Burnham Prebys Medical Discovery Institute, La Jolla, CA, United States

Correspondence: Dmitry A. Rodionov - rodionov@sbpdiscovery.org

*BMC Bioinformatics* 2020, **21(Suppl 20)**: P9

**Background** Production of short-chain fatty acids (SCFAs) by colon microbiota is of utmost importance for human health. Many factors including diet and microbiome composition affect the production of SCFAs by gut microbiota.

**Materials and methods** We developed the Phenotype Profiler tool enabling prediction of metabolic capabilities of human gut microbiome (HGM) to synthesize vitamins, amino acids, SCFAs and utilize carbohydrates based on taxonomic abundance profiles. The tool is utilizing the concept of binary metabolic phenotype assigned to each genome in the reference collection of HGM genomes. We performed metabolic reconstruction of butyrate, propionate, acetate, lactate and formate fermentation pathways in > 2600 microbial genomes using a subsystems-based approach implemented in the SEED database. As result, each reference genome was assigned a binary phenotype reflecting the presence/absence of at least one functional pathway variant. Both for butyrate and propionate synthesis we described 4 alternative pathway variants. The obtained binary phenotypes of reference genomes were used to calculate weighted phenotypes for each mapped amplicon sequence variant (ASV) obtained from metagenomic samples and further calculate Community Phenotype Index (CPI) of each sample as a sum by ASVs of the respective weighted phenotypes multiplied by their relative abundances.

**Results** We collected nine previously published metagenomics datasets obtained in the course of either in vivo (human, mice, rats, piglets), or in vitro fermentation studies and containing both 16S rRNA sequencing data and SCFA metabolomic measurements to investigate if the predicted metabolic potentials (calculated as CPI values) correlate with measured metabolite concentrations. Each 16S dataset was analyzed using the DADA2 plugin from QIIME2. The obtained ASVs were annotated using a multi-taxonomic assignment approach based on similarity to 16S sequences from the RDP database. The Phenotype Profiler tool was applied to the annotated ASV abundance tables to calculate CPI values for SCFAs production in each sample. The obtained CPI values showed the absence of correlation with experimentally measured concentrations of butyrate and propionate in fecal samples from five in vivo studies, which can be explained by highly efficient absorption of SCFAs in the large intestine. In contrast, the CPI values correlate with the level of SCFAs obtained from in vitro bacterial fermentation experiments of fecal inoculum.

**Conclusions** CPI gives a probabilistic estimate of the fraction of community cells possessing a specific metabolic capability. The high concordance between in silico predicted butyrate and propionate production capabilities and their in vitro measured concentrations provide a validation of our phenotype profiling approach.

**Acknowledgments**

This research was supported by the Russian Science Foundation (Grant #19-14-00305).

## P10

### Transcriptomic signatures of seed maturation heterochrony in garden pea (*Pisum sativum* L) accessions

#### Yury V. Malovichko^1,2*^, Oksana Y. Shtark^3^, Ekaterina N. Vasileva^3^, Anton A. Nizhnikov^1,2^, Kirill A. Antonets^1,2^

##### ^1^Laboratory for Proteomics of Supra-Organismal Systems, All-Russia Research Institute for Agricultural Microbiology (ARRIAM), St. Petersburg, 196608, Russia; ^2^Faculty of Biology, St. Petersburg State University, St. Petersburg, 199034, Russia; ^3^Department of Biotechnology, All-Russia Research Institute for Agricultural Microbiology (ARRIAM), St. Petersburg, 196608, Russia

Correspondence: Yury V. Malovichko - yu.malovichko@arriam.ru

*BMC Bioinformatics* 2020, **21(Suppl 20)**: P10

Seed developmental studies remain one of the crucial points in modern molecular plant science. However, the temporal control of embryogenesis and seed maturation remains inconsistently studied. In the present work we used Sprint-2, a rapidly maturing line of garden pea (*Pisum sativum* L.), to study the processes accompanying seed development heterochrony by using whole transcriptome sequencing (RNA-Seq) technology [1]. First, the Sprint-2 seeds of 10 and 20 days after pollination (DAP) were compared in terms of differential gene expression to assess their developmental identity and highlight the processes occurring between these two time points. Apart from the well-known hallmarks of seed maturation, a total of three hundred of mobile element-associated transcripts were found to be upregulated at 20 DAP. Then, the incorporation of external data for two pea lines with an average seed maturation rate [2] revealed that Sprint-2 undergoes developmental retardation at the pre-storage phase of development followed by developmental acceleration after 10 DAP. Consistent with this notion, transcripts associated with storage compound accumulation and acquisition of desiccation tolerance were found to be expressed earlier in Sprint-2. The earlier transition to maturation in Sprint-2 can be partly explained by the premature activation of genes encoding for primary LAFL transcription factors, which were also found to bear strong missense substitutions compared to the two other pea lines. Moreover, at both 10 and DAP Sprint-2 demonstrated an elevated rate of mobile genetic element activity, mostly of retrotransposons of Copia family. The promoted transposon activity may be connected to an altered pattern of DNA methylase genes found in Sprint-2. This includes an earlier onset of *CROMOMETHYLASE* (*CMT*) pathway and an elevated level of *RDM1* and *DRM* genes expression at 20 DAP compared to the two normally maturing accessions. The obtained data indicate that transposable element activity may underlie or rather accompany seed development heterochrony in pea, but further experiments are needed to elucidate this hypothesis.

**Acknowledgments**

This work was financially supported by the Russian Science Foundation (Grant No 17-16-01100).

**References**Malovichko YV, Shtark OY, Vasileva EN, Nizhnikov AA, Antonets KS. Transcriptomic Insights into Mechanisms of Early Seed Maturation in the Garden Pea (Pisum sativum L.). Cells. 2020;9(3):779. Published 2020 Mar 23.Liu N, Zhang G, Xu S, Mao W, Hu Q, Gong Y. Comparative Transcriptomic Analyses of Vegetable and Grain Pea (*Pisum sativum* L.) Seed Development. Front Plant Sci. 2015;6:1039. Published 2015 Nov 25.

## P11

### Genome assembly of microbes by leveraging evolutionary relationships

#### Urmi Shah^1*^, Srikrishna Subramanian^1*^

##### ^1^Bioinformatics Centre, CSIR-Institute of Microbial Technology, Chandigarh 160036, India

Correspondence: Urmi Shah - urmimshah96@gmail.com; Srikrishna Subramanian - krishna@imtech.res.in

*BMC Bioinformatics* 2020, **21(Suppl 20)**: P11

**Background** Microbial genomics has seen rapid improvements in the past decade primarily due to the development of novel algorithms capable of assembling the data generated by a variety of next-generation sequencing technologies, into a high-quality genome. Depending on the sequencing technology, type of libraries, and the complexity of the genome, this has most often resulted in the generation of draft genomes. The completion of these microbial genomes however, have remained a challenge. Even with the advancement of technologies which produce long reads, the cost-effectiveness of short-read technologies has resulted in the deposition of 468,154 (as of December 2019) permanent-draft genomes (i.e., genomes unlikely to be ever completed) in the NCBI database, while the number of complete genomes is only 16,814. The present work aims to develop a computational workflow to improve the quality of these permanent draft genomes using information from complete genomes of evolutionarily related microbes.

**Materials and methods** The complete genome of *Escherichia coli* (NZ_CP027599.1) was selected as the standard assembly for our study. Short reads data sets of varying read lengths (75 bp to 250 bp) were simulated using the programs ART [1], DWGSIM [2], NEAT [3], pIRS [4], and Wgsim [5]. These reads were reference mapped to the standard assembly (NZ_CP027599.1) using BWA [6], Bowtie [7], Novoalign (https://novocraft.com/), and SMALT (https://www.sanger.ac.uk/tool/smalt-0/). Fifteen different genomes from the genus Citrobacter, Enterobacter, Salmonella, Shigella, and Yersinia at varying evolutionary distance to *Escherichia coli* were used as references, for mapping the aforementioned simulated reads. The best resulting assemblies were selected as input for the software GFinisher [8]. De novo assembly of the simulated reads were done using Unicycler [9] for comparison. Comparison of the different assemblies to the complete genome of *Escherichia coli* (NZ_CP027599.1) were made using QUAST [10] and Circos [11].

**Results** Our workflow uses information from multiple-reference genomes to obtain an improved assembly of the simulated reads of *Escherichia coli* (NZ_CP027599.1). It is envisaged that with the increase in the number of complete genomes of a given Genus in the NCBI, the information contained in the genomes of related microbes can be exploited to obtain an assembly with improved contiguity, and with no loss in strain specific information, using the original short-read data from the short read archive.

**Conclusions** A proof-of-concept using simulated short-read data sets of *Escherichia coli* is presented to highlight the improvements in the assembly guided by multiple reference genomes.

**References**Huang W, Li L, Myers JR, Marth GT. ART: a next-generation sequencing read simulator. Bioinformatics. 2012;28(4):593–4.Homer N. (2010). nh13/DWGSIM. GitHub. https://github.com/nh13/DWGSIMStephens ZD, Hudson ME, Mainzer LS, Taschuk M, Weber MR, Iyer RK. Simulating Next-Generation Sequencing Datasets from Empirical Mutation and Sequencing Models. PLoS One. 2016;11(11):e0167047.Hu X, Yuan J, Shi Y, Lu J, Liu B, Li Z, et al. pIRS: Profile-based Illumina pair-end reads simulator. Bioinformatics. 2012;28(11):1533–5.Heng Li. (2011). lh3/wgsim. GitHub. https://github.com/lh3/wgsimLi H, Durbin R. Fast and accurate short read alignment with Burrows-Wheeler transform. Bioinformatics. 2009;25(14):1754–60.Langmead B, Salzberg SL. Fast gapped-read alignment with Bowtie 2. Nat Methods. 2012;9(4):357–9.Guizelini D, Raittz RT, Cruz LM, Souza EM, Steffens MB, Pedrosa FO. GFinisher: a new strategy to refine and finish bacterial genome assemblies. Sci Rep. 2016;6:34963.Wick RR, Judd LM, Gorrie CL, Holt KE. Unicycler: Resolving bacterial genome assemblies from short and long sequencing reads. PLoS Comput Biol. 2017;13(6):e1005595.Gurevich A, Saveliev V, Vyahhi N, Tesler G. QUAST: quality assessment tool for genome assemblies. Bioinformatics. 2013;29(8):1072–5.Krzywinski M, Schein J, Birol I, Connors J, Gascoyne R, Horsman D, et al. Circos: an information aesthetic for comparative genomics. Genome Res. 2009;19(9):1639–45.

## P12

### Reducing redundancy of input data sets to improve inference of transcription factor binding sites

#### Pavel Vychyk*, Yevgeny Nikolaichik

##### Faculty of Biology, Belarusian State University, Minsk, Belarus

Correspondence: Pavel Vychyk - p.vychik@gmail.com

BMC Bioinformatics 2020, 21(Suppl 20): P12

The majority of bacterial genome annotations lack information about transcription factor (TF) binding sites (operators) which control how genomic information is expressed. We are developing the SigmoID application [1] to solve this problem in a highly automated fashion. In brief, the motif discovery algorithm involves analysing 3D crystal structures of TF-operator complexes, finding TFs with the same contacts between operators and DNA-binding domains (CR-tag) and then looking for autoregulatory operator motifs in the promoter regions surrounding the genes encoding these TFs. The success of motif discovery strongly depends on the diversity of promoter region dataset. Assembling appropriate datasets proved to be challenging due to large sizes and rapid expansion of protein databases. In the first step of our pipeline, homologous TFs with CR-tags identical to the one being studied are retrieved. In our experience, this stage proved to be highly unreliable if public phmmer or blastp servers were used. Local searches require fast workstation and maintaining large databases which is undesirable taking into the account the target audience (bench scientists). Also, many thousands of homologous proteins with matching CR-tag are expected for many TFs, while not more than 30–50 are usually required.

We have replaced the problematic TF homologs search step by fast lookup tables. The tables match a CR-tag to IDs of all proteins with this tag. Usage of the PIR representative proteome databases [2] with different co-membership thresholds as sequence source for building lookup tables mostly solved the excessive redundancy problem. The optimal homologs number could often be achieved by simply taking IDs of the proteins from one of the five lookup tables. If homologs number was still excessive, an additional promoter regions clustering step was performed. We have found MeShClust [3] to be the optimal tool at this stage. The efficiency of different clustering approaches and search options was tested by inferring operator motifs for *Escherichia coli* TFs from several protein families. The double clustering approach proved to be the fastest and produced better motifs in some cases as it didn’t have to resort to random selection of sub-optimal promoter regions when their number was excessive. We have also noticed many cases of SigmoID producing “good” operator motifs matching experimental data when such a motif was not present in the RegulonDB database [4] or was incorrect.

The SigmoID v2 software with CR-tag lookup tables for 13 TF families is available for download on Github at https://github.com/nikolaichik/SigmoID.

**References**Nikolaichik Y, Damienikan AU. SigmoID: a user-friendly tool for improving bacterial genome annotation through analysis of transcription control signals. PeerJ. 2016;4:e2056.Chen C, Natale DA, Finn RD, Huang H, Zhang J, Wu CH, et al. Representative proteomes: a stable, scalable and unbiased proteome set for sequence analysis and functional annotation. PloS One. 2011;6:e18910.James BT, Luczak BB, Girgis HZ. MeShClust: an intelligent tool for clustering DNA sequences. Nucleic Acids Res. 2018;46:e83–e83.Gama-Castro S, Salgado H, Santos-Zavaleta A, Ledezma-Tejeida D, Muñiz-Rascado L, García-Sotelo JS, et al. RegulonDB version 9.0: high-level integration of gene regulation, coexpression, motif clustering and beyond. Nucleic Acids Res. 2016;44:D133–43.

